# The influence of childhood maltreatment on substance use among students in Gondar Town, Northwest Ethiopia: the mediating role of social support

**DOI:** 10.3389/fpsyt.2025.1559939

**Published:** 2025-06-17

**Authors:** Angwach Abrham Asnake, Asefa Adimasu Taddese, Mehari Woldemariam Merid

**Affiliations:** ^1^ Department of Epidemiology and Biostatistics, School of Public Health, College of Health Sciences and Medicine, Wolaita Sodo University, Sodo, Ethiopia; ^2^ University of Gondar, Gondar, Amhara, Ethiopia; ^3^ Department of Epidemiology and Biostatistics, Institute of Public Health, College of Medicine and Health Sciences, University of Gondar, Gondar, Ethiopia

**Keywords:** child abuse, child neglect, substance-related disorders, social support, structural equation modeling, mediation analysis, Ethiopia

## Abstract

**Background:**

Childhood maltreatment increases the risk of substance use and substance use disorder (SUD) in adolescence and adulthood, with social support potentially mitigating this relationship. However, research in Ethiopia on mediating factors remains limited. This study uses structural equation modeling (SEM) to evaluate the influence of childhood maltreatment on substance use and the mediating role of social support among students in Gondar Town, Northwest Ethiopia.

**Method:**

A cross-sectional study was conducted from April 18 to May 9, 2023, among 1,235 preparatory and public high school students in Gondar Town who were selected via simple random sampling. Childhood maltreatment was assessed using the Childhood Trauma Questionnaire (CTQ), which yields a total score ranging from 28 to 140. For descriptive analysis, scores were categorized as “none” for values between 25 and 36 or as “some form of childhood maltreatment” for scores above 37. Substance use was measured using the Tobacco, Alcohol, Prescription Medication, and Other Substance Use (TAPS-1) tool. Problematic substance use was defined as any response greater than “never” within the past 12 months. Social support was evaluated using the Multidimensional Scale of Perceived Social Support (MSPSS). Structural equation modeling analyzed the relationships, calculating the mediation proportion.

**Results:**

The median age was 17 years, with 63.24% female participants. Of the students, 85.42% reported a history of childhood maltreatment, 23.48% had problematic alcohol use, and 10.04% had problematic drug use in the past 12 months. Childhood maltreatment significantly increased substance use (β = 1.181, 95% CI (lower, upper): 0.223–1.821). Specific maltreatment types—physical abuse (β = 1.422, 95% CI (lower, upper): 0.590–2.423), sexual abuse (β = 0.653, 95% CI (lower, upper): 0.652–1.320), emotional abuse (β = 2.252, 95% CI (lower, upper): 1.402–4.307), physical neglect (β = 4.101, 95% CI (lower, upper): 1.042–0.904), and emotional neglect (β = 1.513, 95% CI (lower, upper): 0.831–3.059)—were positively associated with substance use. Social support negatively mediated 28.30% of this relationship, reducing the effect of maltreatment on substance use.

**Conclusion:**

Physical abuse, sexual abuse, emotional abuse, physical neglect, and emotional neglect all increase the likelihood of substance use. However, social support mitigates the relationship between childhood maltreatment and substance use. These findings highlight the need for interventions strengthening social support to mitigate the impact of maltreatment on substance use in Ethiopia.

## Introduction

Both childhood maltreatment and substance use disorder (SUD) are significant public health concerns with profound implications, particularly in low- and middle-income countries (1, 2). Substance use, defined as the improper or risky consumption of psychoactive substances including alcohol, tobacco, prescription medications, and illicit drugs, contributes to a global burden of 35 million people experiencing risky drug use or dependence in 2022, as reported by the World Health Organization (WHO) (3). In Sub-Saharan Africa (SSA), substance use remains a growing challenge, with limited regional data indicating high prevalence rates among youth; approximately 31.2%–44.6% of adolescents in SSA engage in problematic alcohol or drug use (4). Among university students in Addis Ababa, Ethiopia, 56.8% consumed alcohol, 40.2% used khat, 14.1% smoked cigarettes, and 16.2% actively used illicit drugs (5). In Bahir Dar, Ethiopia, the prevalence of problematic substance use was 55.8% (6). These figures highlight the urgent need for targeted interventions in resource-limited settings like Ethiopia.

Adolescents and adults with SUD often have a history of childhood maltreatment, encompassing physical abuse, sexual abuse, emotional abuse, physical neglect, and emotional neglect (7, 8). Research has indicated that such maltreatment types disrupt emotional regulation and increase vulnerability to maladaptive coping mechanisms, including substance use (9). Moreover, childhood maltreatment can erode social support systems, which are critical protective factors (10, 11). Higher levels of social support encompassing family, peers, and other significant sources are associated with reduced substance use, potentially buffering the adverse effects of maltreatment (8). This protective role highlights social support as a potential mediator, an area needing further exploration, particularly in less-developed contexts.

Previous studies have often combined different types of childhood maltreatment into cumulative scores, limiting the ability to examine their independent effects (12). This approach overlooks the distinct impacts of, for example, emotional neglect versus physical abuse, which may differentially influence substance use outcomes. Additionally, many studies have treated latent variables like social support as observed, reducing analytical depth (13). Most investigations rely on classical models to assess direct effects, with scant attention to mediating pathways in settings like Ethiopia (14). Thus, this study investigated the role of social support in the relationship between childhood maltreatment and substance use using structural equation modeling (SEM) among adolescents and adults of Gondar Town. By focusing on a less-developed country context, this research aimed to provide evidence for tailored interventions to mitigate SUD among maltreated youth.

## Materials and methods

### Research design and period

This study employed an institution-based cross-sectional design to assess the relationship between childhood maltreatment, social support, and substance use. This design was chosen to capture data at a specific point in time, allowing for an in-depth examination of associations between key variables in a real-world setting. The study was conducted from April 18 to May 9, 2023, among students in public high schools and preparatory schools in Gondar Town. Gondar Town serves as the capital of the North Gondar Zone in the Amhara Regional State. This research was carried out as an independent study to contribute to the growing body of knowledge on childhood maltreatment’s influence among adolescents and young adults in Ethiopia.

### Inclusion and exclusion criteria

The source population consisted of all Gondar Town public high school and preparatory school students, while the study population consisted of all students who registered for the second semester of the 2023 academic year. This survey included all Gondar Town public high and preparatory school students registered for the second semester of the 2023 academic year. Students from night prep schools and night high schools were not included in the study.

### Sampling

**Figure 1:**
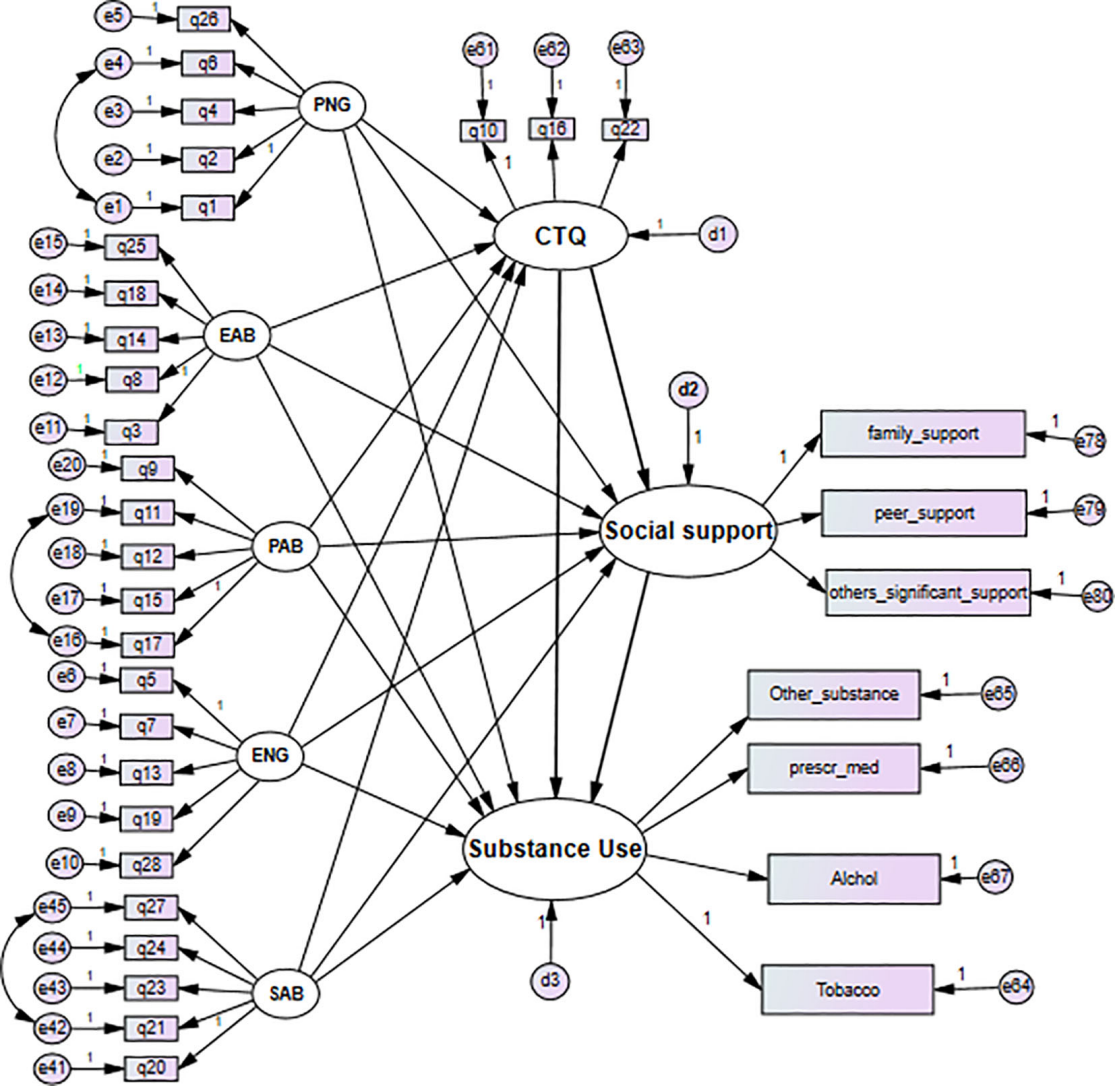
A hypothesized causal pathway shows the effect of childhood maltreatment and social support on substance use. Key: CM, childhood maltreatment; PA, physical abuse; EN, emotional neglect; PN, physical neglect; SA, sexual abuse; EA, emotional abuse. A simple random sampling technique was used to select study participants. There are 12 public high schools and preparatory schools, with an overall enrolment of 23,524. The list of student ID numbers served as a sampling frame for the computer-generated random number selection process.


This study used the practical recommendation on sample size for structural equation modeling a 10:1 ratio of a sample size to the number of free parameters (15). According to the hypothetical model (**Figure 1**), there are six covariances between latent variables, 35 observed endogenous variables, 27 loadings (because seven of them fixed to one to give latent measurement scale), 35 indicators’ error term variance, three latent variables’ error variance, and 13 path coefficients, resulting in 119 parameters that need to be estimated in total.

n=119∗10+(119∗10∗0.05)=1,250

Therefore, by adding a 5% non-respondent rate, our final sample size should be 1,250. The required sample size was estimated to be 1,250. The current analysis took into account the entire sample of 1,279 individuals, as this study was carried out in support of another research objective, and the full sample was utilized for the psychological wellbeing analysis.

### Data collection tool

#### Childhood Trauma Questionnaire

The short version of the Childhood Trauma Questionnaire (CTQ-SF) (16) was used in this study to assess childhood maltreatment, including physical abuse, sexual abuse, emotional abuse, physical neglect, and emotional neglect. This 28-item questionnaire, originally in English and translated into Amharic for this study, includes five subscales (five items each) and three Minimization/Denial Scale items to detect false-negative reporting. The CTQ-SF demonstrates strong psychometric properties, with test–retest reliability of 0.85 and internal consistency (Cronbach’s alpha) ranging from 0.63 to 0.96 among adolescents and adults (17–20). The Childhood Trauma Questionnaire has a total of 28 to 140 scores; for descriptive purposes, childhood maltreatment was categorized as none if the score is 25 to 36 or as some forms of childhood maltreatment if it is greater than 37 (21).

#### Tobacco, Alcohol, Prescription Medication, and Other Substance Use tool

The Tobacco, Alcohol, Prescription Medication, and Other Substance Use (TAPS-1) tool (22) measured substance use, including tobacco, alcohol, prescription medications, and other drugs (e.g., marijuana, cocaine, and heroin). This tool, originally in English and translated into Amharic, uses a 5-point Likert scale (0 = never, 1 = less than monthly, 2 = monthly, 3 = weekly, and 4 = daily) to assess use over the past 12 months. Problematic use was defined as any response greater than “never”. Validation studies have confirmed its effectiveness in identifying moderate- to high-risk use for tobacco, alcohol, and prescription opioids (23, 24). Cigarette smoking, alcohol consumption, non-medical use of medications, and other substance use were assessed using the TAPS-1 tool. Any response greater than “never” within the past 12 months was considered indicative of problematic use (24). Non-medical use of prescription medications refers to use “just for the feeling”, in amounts greater than prescribed, or use of medications not prescribed to the respondent behaviors that are categorized as prescription drug misuse and are classified under substance use.

#### Multidimensional Scale of Perceived Social Support

The Multidimensional Scale of Perceived Social Support (MSPSS) was employed to evaluate social support, encompassing peer, family, and other significant support. Having four items each, each dimension has a Likert scale from 1 to 7. The mean scale score is divided into three categories: low support (scores of 1 to 2.9), moderate support (scores of 3 to 5), and great support (scores of 5.1 to 7) (25).

### Data collection

A self-administered, structured questionnaire was used to gather data. Four skilled data collectors with a public health officer first degree were given the task of gathering data. Based on the recommended appropriate sample size for the pilot study, 200 public high and preparatory school students from Negus Teklehaimanot Secondary and High School in Debre Markos Town participated in an external pilot study to verify the processes of data collection, administration, and analysis (26). The pilot study additionally enabled us to assess how different participants understood each questionnaire, and we were able to make any necessary modifications for the final study based on their comments. For respondents who were unavailable when the data were collected, repeated trials were taken into consideration. Data were entered into EpiData after being coded. Missing value management was taken into account after data entry.

### Model building and analysis

The completeness of the data was manually verified before analysis. The dataset was entered into EpiData version 3.6, and initial data handling was conducted and subsequently exported to STATA version 17 for further statistical analysis. SEM was conducted using AMOS version 24. Descriptive and summary statistics were presented in text, figures, and tables. Reliability and validity were assessed using Cronbach’s alpha (>0.7) and average variance extracted (AVE) or composite reliability (>0.5) (27, 28). Mardia’s coefficients confirmed non-normal data distribution, addressed using 1,000-sample bootstrap maximum likelihood estimation. The Mahalanobis distance identified 76 outliers (p< 0.001), which were re-included after verifying data entry accuracy. Unstandardized coefficients with 95% confidence intervals and 5% significance levels were reported.

### Ethical considerations

All methods were conducted in compliance with national regulations and the Declaration of Helsinki. Ethical approval was obtained from the Institutional Review Board (IRB) of the University of Gondar College of Medicine and Health Sciences (Reference No. IPH/2489/08/2023). Written informed consent was obtained from all participants, and for those under 18, consent was obtained from their parents or legal guardians, along with participant assent.

To ensure privacy and confidentiality, all data were stored securely in a locked cabinet accessible only to the investigators. Personal identifiers were not included in the dataset, and the collected information was used exclusively for research purposes. Given the sensitivity of childhood maltreatment and substance use, participants were informed of their right to withdraw at any time without consequences. Additionally, referral information for psychological and social support services was made available; however, no participants reported experiencing distress or requiring further assistance during the study.

## Results

### Socio-demographic characteristics of the respondents

**Figure 2:**
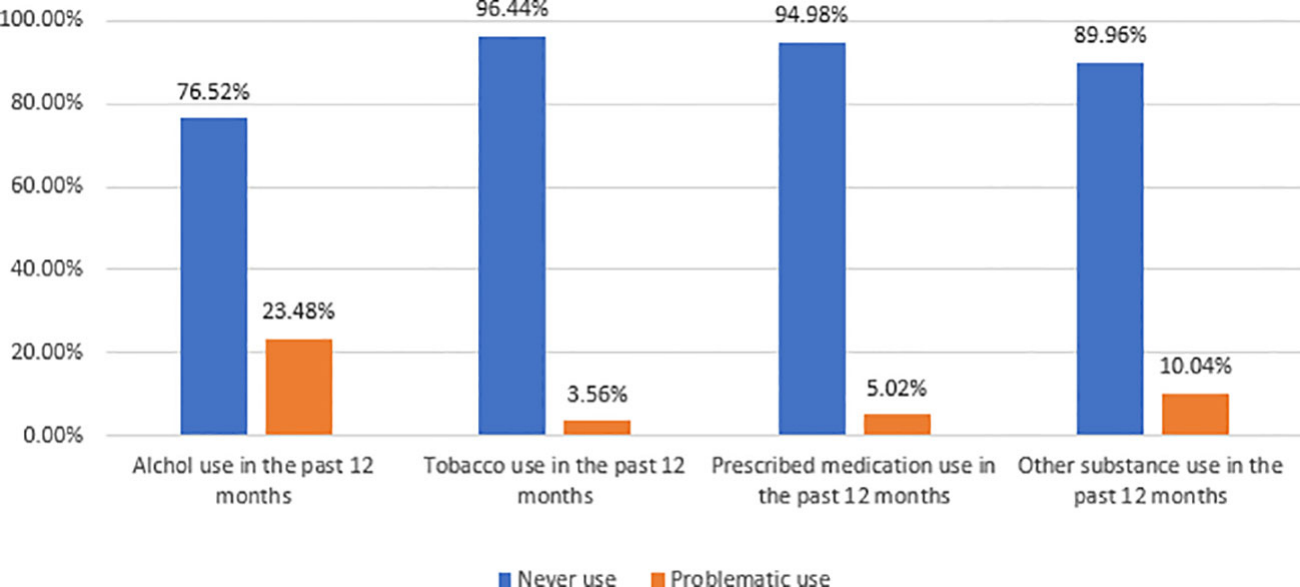
Magnitude of substance use among high and preparatory school students in Gondar Town, Northwest Ethiopia, 2023 (n = 1,235).

Out of 1,309 randomly selected students, a total of 1,235 respondents completed the questionnaires, resulting in a response rate of 94.3%. A majority of 781 (63.24%) participants were identified as female. The median age of the respondents was 17 years (IQR, 3 years). A majority of 991 (80.24%) respondents reported that both their mother and father served as their primary caregivers. Approximately 241 (19.51%) of the participants reported low levels of peer support, 205 (16.60%) experienced low levels of family support, and 197 (15.95%) reported low levels of other significant support (**Table 1**). Approximately 290 (23.48%) of the respondents had problematic alcohol use in the last 12 months. Of the students, 85.42% reported a history of childhood maltreatment to varying degrees, 290 (23.48%) had problematic alcohol use, and 124 (10.04%) had problematic drug use in the past 12 months (**Figure 2**).

**Table 1:** Socio-demographic and psychosocial characteristics of high and preparatory school students in Gondar Town, Northwest Ethiopia, 2023 (n = 1,235).

Characteristics	Category	Frequency (%)
Sex	Male	454 (36.76%)
	Female	781 (63.24%)
Caregiver	Mother alone	141 (11.42%)
	Father alone	11 (0.89%)
	Both father and mother	991 (80.24%)
	Parent with stepparent	39 (3.16%)
	Others	53 (4.29%)
Family support	Low	205 (16.60%)
	Moderate	341 (27.61%)
	High	689 (55.79%)
Peer support	Low	241 (19.51%)
	Moderate	365 (29.55%)
	High	629 (50.93%)
Other significant support	Low	197 (15.95%)
	Moderate	453 (36.68%)
	High	585 (47.37%)

### Correlation between childhood maltreatment, social support, and substance use

Childhood maltreatment had a moderate correlation (0.5 > r > 0.3, p< 0.01) with social support and substance use. A weak correlation (r< 0.3, p< 0.01) was observed between social support and substance use (**Table 2**).

**Table 2:** Spearman’s correlation between childhood maltreatment, social support, and substance use of high and preparatory school students in Gondar Town, Northwest Ethiopia 2023 (n = 1,235).

Variables	Childhood maltreatment	Social support	Substance use
Childhood maltreatment	1.000		
Social support	−0.38^**^	1.000	
Substance use	0.34^**^	−0.113^**^	1.000

^**^ p< 0.01.

### Measurement model

**Figure 3:**
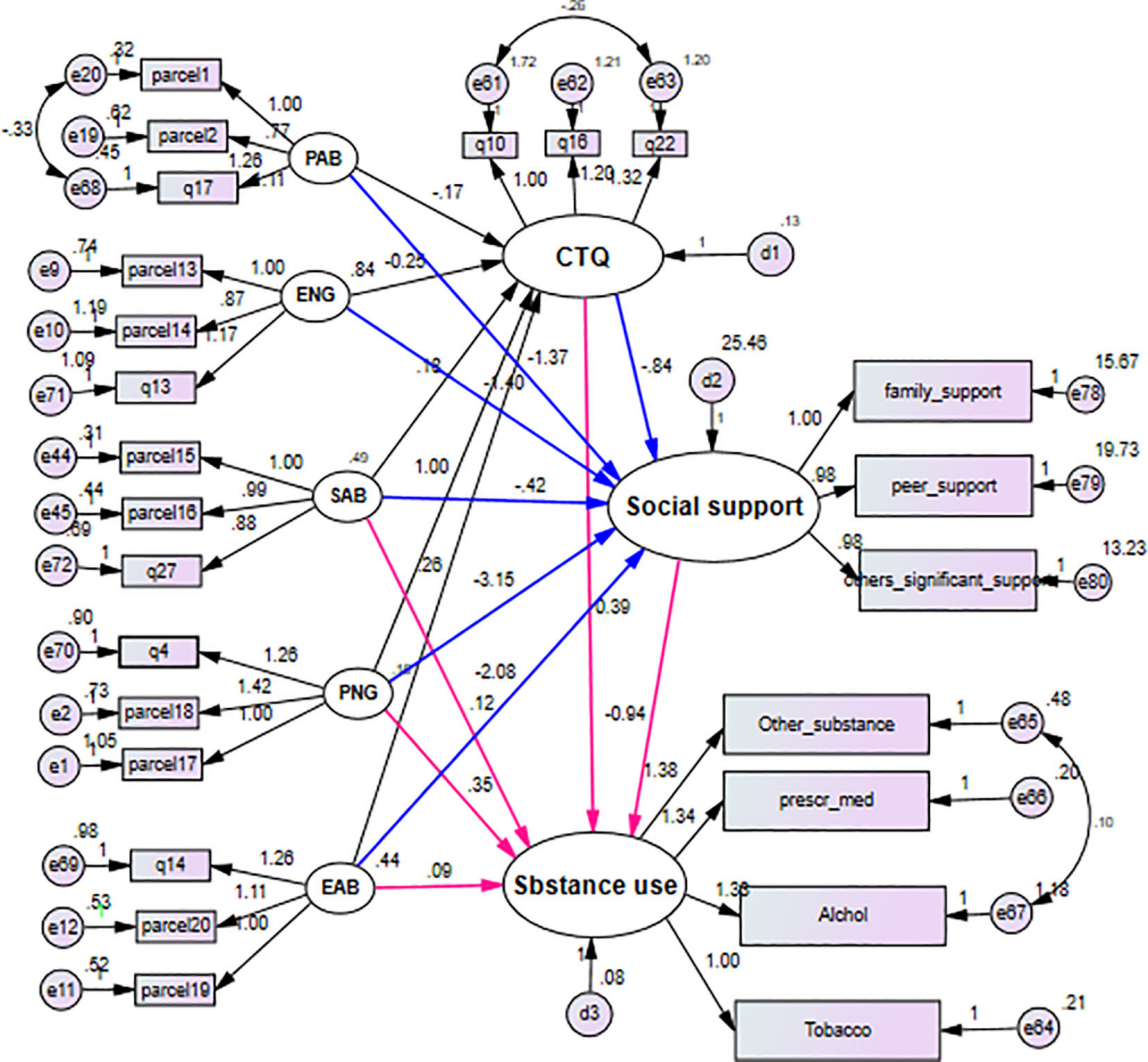
SEM diagram with unstandardized coefficient showing the effect of childhood maltreatment on substance use among high and preparatory school students of Gondar Town, Northwest Ethiopia, 2023. SEM, structural equation modeling.

The measurement model in **Figure 3** shows how latent variables are measured through observed variables, using a confirmatory factor analysis model for grouping multiple indicators into some constructs (29, 30). Overall fit indices were satisfactory [root mean square error of approximation (RMSEA) = 0.05, goodness-of-fit index (GFI) = 0.91, comparative fit index (CFI) = 0.93].

The AVE for emotional neglect, physical neglect, sexual abuse, emotional abuse, and total childhood maltreatment ranged from 0.41 to 0.49, below the 0.5 threshold. However, composite reliability supported adequate convergent validity despite variance explained by error (**Table 3**).

**Table 3:** Reliability and validity of the measurement model with indices.

Constructs and indicators	Factor loading	Cronbach’s alpha	Composite reliability	AVE
Physical abuse		0.83	0.88	0.71
Parcel1	0.92			
Parcel2	0.71			
Q17	0.88			
Emotional neglect		0.72	0.72	0.47
Parcel13	0.73			
Parcel14	0.60			
Q13	0.71			
Sexual abuse		0.73	0.74	0.49
Parcel15	0.80			
Parcel16	0.70			
Q27	0.58			
Physical neglect		0.53	0.66	0.41
Parcel17	0.83			
Parcel18	0.49			
Q4	0.55			
Emotional abuse		0.51	0.72	0.46
Parcel19	0.70			
Parcel20	0.71			
Q14	0.62			
Global measures of CM		0.51	0.71	0.46
Q10	0.74			
Q16	0.61			
Q22	0.67			
Social support		0.62	0.82	0.61
Family support	0.86			
Peer support	0.64			
Other significant support	0.81			
**TAPS**		0.60	0.68	0.50
Alcohol	0.51			
Tobacco	0.57			
Prescription medications	0.72			
Other substances	0.54			

AVE, average variance extracted; CM, childhood maltreatment; TAPS, Tobacco, Alcohol, Prescription Medication, and Other Substance Use.

### The effect of childhood maltreatment on substance use among adolescents and adults in Gondar Town

**Figure 4:**
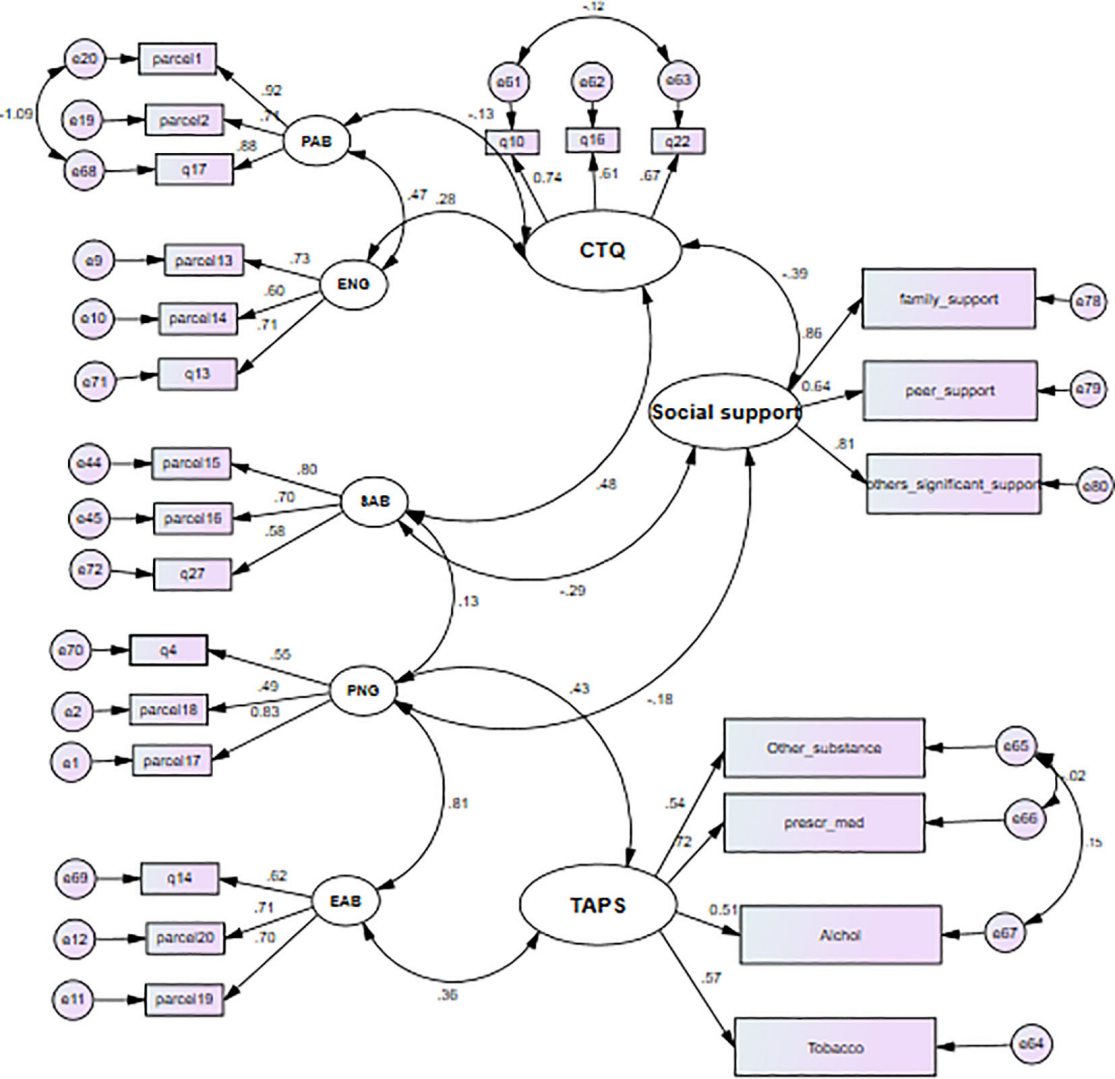
Measurement model diagram with standardized coefficient.

The final model, integrating structural (relationships among latent variables) and measurement components (relationships between latent variables and items), is presented in **Figure 4** and **Table 4**. All path coefficients were statistically significant at an alpha level of 0.05. The model fit was optimal [χ^2^/df = 1.76, p< 0.001, RMSEA = 0.053, Tucker–Lewis Index (TLI) = 0.90, CFI = 0.93], comprising 25 indicator variables (10 parcels of 20 items), five unobserved exogenous variables, and three unobserved endogenous variables.

**Table 4:** Unstandardized estimate shows the effect of childhood maltreatment on substance use of high and preparatory school students in Gondar Town, Northwest Ethiopia 2023 (n = 1,235).

Variables	Direct effect [95% CI (lower, upper)]	Indirect effect [95% CI (lower, upper)]	Total effect [95% CI (lower, upper)]
DV: Social support			
Physical abuse	−1.371 (−2.121, −0.775) ^*^	—	−1.371 (−2.121, −0.775) ^*^
Physical neglect	−3.150 (−5.716, −1.113) ^*^	—	−3.150 (−5.716, −1.113) ^*^
Emotional abuse	−2.075 (−3.191, −1.030) ^*^	—	−2.075 (−3.191, −1.030) ^*^
Emotional neglect	−1.396 (−2.282, −0.491) ^*^	—	−1.396 (−2.282, −0.491) ^*^
Sexual abuse	−0.420 (−1.342, −0.465) ^*^	—	−0.420 (−1.342, −0.465) ^*^
Global measures of CM	−0.840 (−3.542, −2.278) ^*^	—	−0.840 (−3.542, −2.278) ^*^
DV: Substance use			
Social support	−0.94 (−2.431, −0.355) ^*^		−0.94 (−2.431, −0.355) ^*^
Physical abuse	—	1.422 (0.590, 2.423) ^*^	1.422 (0.590, 2.423) ^*^
Emotional neglect	—	1.513 (0.831, 3.059) ^*^	1.513 (0.831, 3.059) ^*^
Emotional abuse	0.092 (0.039, 0.181) ^*^	2.160 (1.564, 3.947) ^*^	2.252 (1.402, 4.307) ^*^
Physical neglect	0.350 (0.155, 0.596) ^*^	3.751 (1.038, 4.250) ^*^	4.101 (1.042, 0.904) ^*^
Sexual abuse	0.116 (0.064, 0.225) ^*^	0.537 (0.604, 1.052) ^*^	0.653 (0.652, 1.320) ^*^
Global measures of CM	0.391 (0.183, 0.659) ^*^	0.790 (0.036,1.182) ^*^	1.181 (0.223, 1.821) ^*^

DV, dependent variable; CM, childhood maltreatment.

^*^p-Value<0.05.

Physical abuse had an indirect positive effect (β = 1.422, 95% CI (lower, upper) (0.590, 2.423)) on substance use. Sexual abuse had a direct positive effect (β = 0.116, 95% CI (lower, upper) (0.064, 0.225)) and an indirect positive effect (β = 0.537, 95% CI (lower, upper) (0.604, 1.052)) on substance use, giving a total positive effect of β = 0.653, 95% CI (lower, upper) (0.652, 1.320). Childhood maltreatment had a direct positive effect (β = 0.391, 95% CI (lower, upper) (0.183, 0.659)) and an indirect effect (β = 0.790, 95% CI (lower, upper) (0.036, 1.182)) on substance use, yielding a total positive effect of β = 1.181, 95% CI (0.223, 1.821). These indicate that both social support and global measures of childhood maltreatment mediated the relationship between all forms of maltreatment and substance use outcomes. The total effect of physical abuse, sexual abuse, emotional abuse, physical neglect, and emotional neglect was mediated by global measures of childhood maltreatment and social support. The indirect standardized effect of childhood maltreatment on substance use through social support was β = 0.075, p< 0.05. The mediated effect of physical abuse through social support alone was β = 0.199, p< 0.05, and the mediated effect of physical abuse through global childhood maltreatment and social support was β = 0.020, p< 0.05 (**Tables 4**, **5**).

**Table 5:** Summary of the standardized effect for all hypothesized pathways.

Pathway	Effect of CM on SU through mediators	Indirect effect
PAB→CM→SS→SU	0.020	
PAB→SS→SU	0.199	0.219
ENG→CM→SS→SU	0.027	
ENG→SS→SU	0.183	0.210
EAB→CM→SS→SU	0.020	
EAB→SS→SU	0.191	0.211
PNG→CM→SS→SU	0.046	
PNG→SS→SU	0.174	0.220
SAB→CM→SS→SU	0.015	
SAB→SS→SU	0.057	0.057
CM→SS→SU	0.075	0.075

PAB, physical abuse; PNG, physical neglect; ENG, emotional neglect; SAB, sexual abuse; EAB, emotional abuse; CM, childhood maltreatment; SS, social support; SU, substance use.

The mediating variable social support had a negative direct effect (β = −0.83, p< 0.05) on substance use. The standardized direct effect of overall childhood maltreatment on substance use was β = 0.19, p< 0.05, with an indirect effect of β = 0.075, p< 0.05, yielding a total effect of β = 0.265, p< 0.05 (**Table 4**).

The mediation proportion (MP) is given as follows: $\;\text{MP}=\frac{\text{indirect effect}}{\text{ direct effect}+\text{indirect effect}}*100\%=28.30\%$ .

The mediation proportion of social support on the association between overall childhood maltreatment and substance use was 28.30%.

## Discussion

This study utilized SEM to effectively account for measurement errors while exploring the direct and mediated effects of childhood maltreatment on substance use through social support. The results indicated that except for physical and emotional abuse, all other types of childhood maltreatment had a direct and significant influence on substance use. Although physical and emotional abuse did not directly impact substance use, their effects were mediated by social support. Notably, social support played a crucial role, mediating 28.30% of the relationship between overall childhood maltreatment and substance use. These findings highlight the essential buffering effect of social support in reducing the negative consequences of childhood maltreatment on substance use.

The current study unequivocally demonstrated, consistent with prior research, that childhood maltreatment was directly associated with an increased risk of substance use among school-aged adolescents and adults (8, 31–33). Both directly and through social support, sexual abuse had increased effects on substance use. This finding is consistent with a study carried out in the United States (34). As physical neglect increases, substance use also increases, resulting from both a direct effect and a mediated effect through social support. This finding is consistent with studies conducted in the United States (35, 36). Emotional abuse had positive direct and indirect effects on substance use. This aligns with previous research (37, 38). Emotional neglect had an indirect effect on substance use, mediated through social support. This finding supports studies conducted in Germany (36) and Turkey (39). A possible explanation for this is that children who experience abuse and neglect may have more difficulty managing their emotional responses, which could make it harder for them to cope with distressing situations in healthy ways (40). As a result, maltreated individuals may turn to substances as a coping mechanism to manage the psychological and emotional pain caused by maltreatment. This aligns with the broader literature, which suggests that childhood maltreatment can disrupt emotional regulation and contribute to maladaptive coping strategies, including substance use (41). The association between childhood maltreatment and substance use was mediated by social support (8, 42). This finding is convergent with studies from the Midwestern metropolitan area (10) and Ireland (11). This indicates social support serves as a buffer in the relationship between childhood maltreatment and substance use. The possible explanation for this may be that individuals with maltreatment seem less emotionally mature than their peers because childhood maltreatment could make it harder to grow a trusting relationship with their own emotions. Therefore, those with a history of childhood maltreatment may have limited access to the necessary supportive networks or resources to buffer these adverse effects. Moreover, this results in a higher frequency of emotional problems, and to cope with this stress, they may turn to substances.

However, while these patterns are common across different international settings, it is essential to recognize the local factors that may influence the relationship between maltreatment and substance use in Ethiopia. For example, young people in Ethiopia may face unique challenges, such as limited access to mental health services, cultural stigmas surrounding both substance use and abuse, and the availability of certain substances. These contextual differences can shape how childhood maltreatment impacts substance use and complicate efforts to provide interventions that work in other settings.

The findings of this study not only enhance the international understanding of the relationship between childhood maltreatment and substance use but also underscore the importance of context-sensitive interventions. While international benchmarks offer valuable comparisons, it is crucial for future research and intervention programs to consider the unique challenges faced by Ethiopian youth. Policies should be adapted to address local needs while also drawing on global best practices to ensure their effectiveness. Moreover, the results emphasize the critical role of social support in mitigating the negative consequences of childhood maltreatment on substance use. These findings suggest that interventions and prevention strategies must prioritize the promotion of mental health and the strengthening of social support networks to prevent substance use among maltreated children. It is essential for future programs to not only focus on direct substance use prevention but also address the emotional and psychological impact of maltreatment particularly in settings like Ethiopia, where access to mental health resources may be limited.

### Strengths and limitations of the study

To the best of our knowledge, this is the first study in Ethiopia to investigate the relationships between childhood maltreatment and substance use mediated by social support among high school and preparatory school students. Childhood maltreatment was assessed using the CTQ, a standardized and validated tool applicable in both developed and developing countries. Additionally, this study employed SEM, which allowed for the simultaneous assessment of both direct and indirect effects of multiple predictors on outcome variables, enhancing the robustness of the findings. Despite these strengths, the study has certain limitations. The sample was restricted to adolescents and adults attending public schools, which may limit the generalizability of the findings to the broader population, including those in private schools or out-of-school adolescents. A significant limitation is the reliance on self-reported data, which may be subject to recall bias or social desirability effects. This could affect the accuracy of prevalence estimates and the strength of observed associations. Future studies should consider adopting longitudinal designs to establish causality and reduce bias over time.

## Conclusion

This study underscores the significant relationship between childhood maltreatment, substance use, and the role of social support in mitigating these effects. The findings suggest that social support plays a critical role in buffering the adverse consequences of childhood maltreatment on substance use. These results provide valuable insights that can inform the development of interventions and prevention strategies aimed at addressing the complex issue of substance use among adolescents and young adults who have experienced maltreatment. While this study emphasizes the importance of social support in this context, it is important to note that other factors, such as direct substance use prevention and emotional and psychological support, must also be considered in developing comprehensive strategies.

## Data Availability

The raw data supporting the conclusions of this article will be made available by the authors, without undue reservation.
